# Pediatric intranasal lobular capillary hemangioma: a case report and review of the literature

**DOI:** 10.1093/jscr/rjae119

**Published:** 2024-04-26

**Authors:** Jood K Alotaibi, Osama Ali Alotayf, Mohammed Jihad Almahdi, Mohammed Halwani

**Affiliations:** College of Medicine, Imam Abdulrahman Bin Faisal University, Dammam 32210, Eastern Province, Saudi Arabia; College of Medicine, Jazan University, Jazan 82511, Jazan Province, Saudi Arabia; Department of Otolaryngology - Head and Neck Surgery, King Saud Bin Abdulaziz for Health Sciences, National Guard Riyadh, Saudi Arabia; Pediatric Otolaryngology Division, King Saud Bin Abdulaziz for Health Sciences, King Abdullah Specialized Children’s Hospital, National Guard Riyadh, Riyadh 12211, Riyadh Province, Saudi Arabia

**Keywords:** lobular capillary hemangioma, epistaxis, obstruction, surgical excision

## Abstract

Lobular capillary hemangioma is a benign lesion commonly affecting the head and neck region. However, in children, it is commonly seen in the buccal mucosa, gingiva, and the tongue, but its presence in the nasal cavity is less frequent. The most common symptoms of nasal hemangiomas are epistaxis and nasal obstruction. However, we present a case of a thirteen-year-old male having intranasal lobular capillary hemangioma with a 2-day history of left-sided epistaxis. The diagnosis is confirmed by histological examination, and the treatment is done by endonasal endoscopic excision of the hemangioma with cauterization of the feeding vessel has performed to remove the lesion completely. Moreover, the diagnosis of lobular capillary hemangioma must always be kept in mind when discussing the differential diagnosis of a bleeding mass within the nasal cavity, even though it is a rare condition and surgical excision is still the preferred first-line treatment.

## Introduction

Hemangiomas are benign lesions caused by abnormal blood vessel growth, commonly affecting the skin of the head and neck region and mucus membranes of oral cavity [1, 2]. Rarely, they affect the nasal cavity [2]. Hemangiomas typically appear as painless nasal masses with intermittent epistaxis or rhinorrhea [2]. The exact etiology remains unknown, but they are often seen during pregnancy, in patients using oral contraceptives, or in those with a history of trauma [2]. Radiological imaging, including computed tomography (CT) and magnetic resonance imaging (MRI), helps characterize intranasal hemangiomas. The distinctive radiological features, aid in distinguishing these vascular lesions from other nasal pathologies such as nasal polyps, angiofibroma, and inflammatory masses [2]. The occurrence of rapidly growing intranasal masses in children with nasal obstruction and epistaxis is an alarming clinical sign [2]. Moreover, the diagnosis should be confirmed by histological examination and the treatment of lobular capillary hemangioma (LCH) involves surgical excision [2]. Reporting such cases is important due to the rarity of nasal hemangiomas in pediatrics. This case report illustrates a 13-year-old patient with acute-onset epistaxis and intermittent left nasal obstruction which was later confirmed to have LCH.

## Case presentation

**Figure 1:**
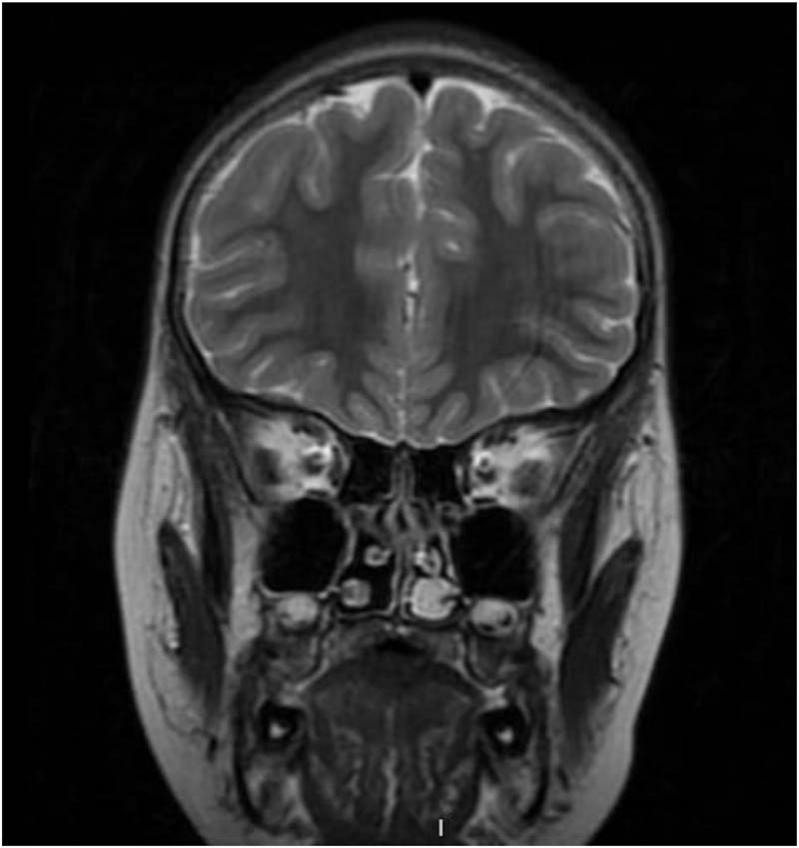
Pre-operative coronal T2-weighted brain MRI showing high signal intensity left-sided intra-nasal lesion.

**Figure 2:**
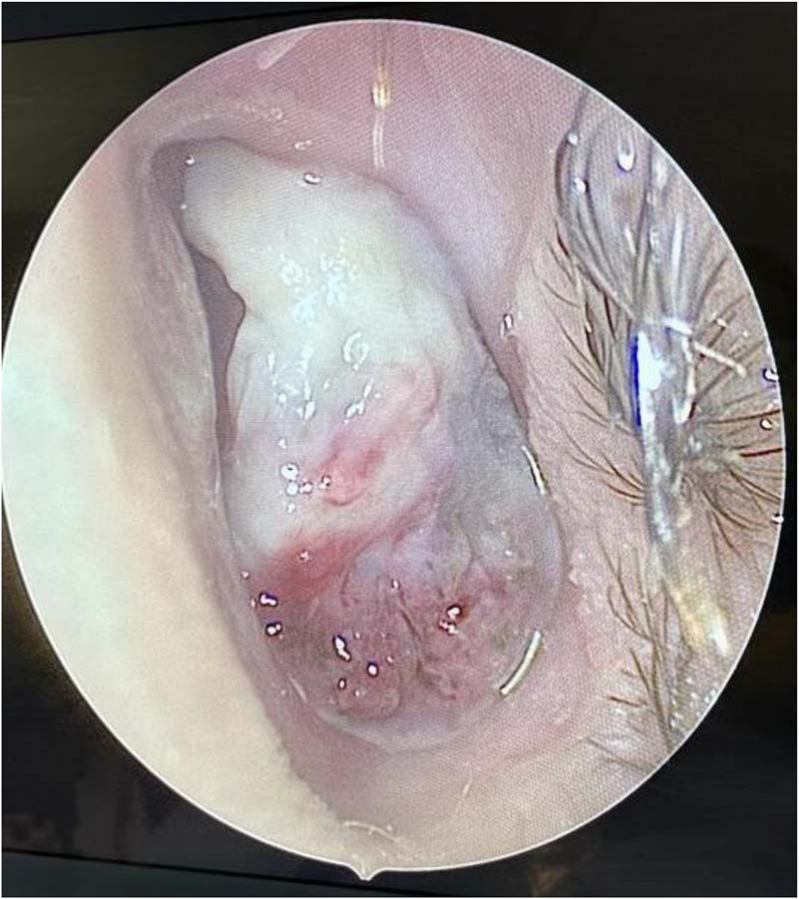
Intra-operative endoscopic findings of the left nasal cavity, showing a vascularized mass occupying the left nasal cavity.

We present a case of a medically free 13-year-old male, who presented to the emergency department with a left-sided epistaxis for 2 days. Notably, the episodes of epistaxis resolve spontaneously after 5 to 10 min, without applying pressure. Also, the patient complained of on and off left-sided nasal obstruction, and denied history of dizziness, loss of consciousness, and choking. Upon physical examination, the patient was vitally stable, looking well, not cyanosed, nor having cervical lymph node enlargement. Nasal speculum examination showed left intra-nasal mass obstructing the cavity. Routine laboratory investigations yielded a hemoglobin (Hgb) level 0f 141g/L, white blood cell count of 7.30 K/μl, INR of 1.02, prothrombin time (PT) of 11.40 s, and partial thrombin time (PTT) of 28.30 s. Initially, the patient was radiologically investigated by paranasal sinuses (PNS) computed tomography (CT) which demonstrated an enhancing lesion involving the left nasal cavity. An MRI was performed demonstrating an oval-shaped mass filling the anterior nasal cavity, the lesion was measuring ~3 × 1.1 × 2.5 cm in anteroposterior, transverse, and craniocaudal dimension, respectively (Fig. 1). Intra-operative nasal cavity along with postnasal space examination was decided to be performed along with left nasal mass excisional biopsy under general anesthesia. Intraoperative examination of the nasal cavity using a zero-degree rigid endoscope showed a fragile left nasal mass (Fig. 2). The lesion was bleeding heavily; thus, cauterization was performed to control bleeding before taking the biopsy. The patient had an uneventful postoperative course. Histopathological evaluation showed the characteristic polypoidal lesion composing of capillary proliferation in a background of fibromyxoid stroma and granulation tissue, arranged in a lobular pattern.

## Discussion

Lobular capillary hemangioma, previously known as pyogenic granuloma, is a benign vascular proliferative lesion of an unknown origin with a characteristic histological lobular pattern [3]. Notably, the description of this lesion as a pyogenic granuloma is now considered a misnomer since the lesion does not have an infectious or granulomatous origin [4]. This condition primarily affects the head and neck region and accounts for 7% of benign pediatric head and neck tumors [2]. Nasal LCH represents 15%–20% of the cases and typically occurs in the third to fifth decade of life [2]. A retrospective study of 178 infants and children with LCH showed a male predominance, accounting for 60.1% of cases [3].

The exact etiology of nasal LCH is still unknown, however, several theories have been proposed. These include viral oncogenesis, arteriovenous malformation, elevated hormone levels, and trauma from picking or packing the nose [4]. The latter is the most frequently reported underlying etiology in the literature, leading to the development of an anterior nasal septum lesion at the level of kisselbach triangle or the nasal turbinates [4].

The prevalence of intranasal hemangiomas remains relatively low, but their impact on pediatric patients cannot be underestimated. LCH often presents with rapidly growing nasal mass associated with recurrent unilateral epistaxis, nasal obstruction, hyposmia or anosmia, headache, facial pain, and purulent rhinorrhea [5]. The clinical presentation can range from mild to severe nasal obstruction, potentially to feeding difficulties, sleep disturbances, and failure to thrive [2]. Thus, ruling out juvenile nasopharyngeal angiofibroma, foreign bodies, dermoid cysts, paraganglioma, angiosarcoma, and angiomatous polyps via imaging modalities and histopathological evaluation is crucial [3]. An endoscopic nasal examination can reveal a hyper-vascularized mass in the nasal cavity, which is supported by imaging findings [1.4]. CT scans are necessary to exclude intracranial extensions, showing a unilateral soft tissue density nasal mass obscuring the cavity [5]. T2-weighted images will demonstrate the characteristic high signal intensity mass surrounded by flow voids [5].

Based on our literature review, 17 pediatric patients with nasal hemangioma were identified, ranging from 45 days old to 14 years old with an average age of 9 years old (Table 1). Most of these patients were males (10 patients) representing 59% of the total cases, whereas the remaining were females (7 patients). Of the previously reported 17 cases, the most frequent symptoms were related to the hypervascularized characteristic of the lesion. Those patients reported the occurrence of unilateral recurrent epistaxis and nasal obstruction. In this case report, our patient had an unusual acute-onset of these symptoms, in contrast to the reported case where they experienced a gradual onset of their clinical complaints. As in our case, the management of intranasal LCH is achieved via the minimally invasive transnasal endoscopic excision of the lesion along with cauterization to establish hemostasis [5].

**Table 1:** Summarized cases of pediatric intranasal lobular capillary hemangioma

Reference	Age	Gender	Clinical Presentation	Topography	Histopathology	Management
Al Washahi MK *et al.* [4]	12 years-old	Female	Epistaxis	Inferior turbinate	Lobular capillary hemangioma	Endoscopic excision
Karagama YG *et al.* [5]	8 years-old	Male	Nasal obstruction Nasal discharge Epistaxis	The floor of the nasal vestibule	Lobular capillary hemangioma	Elliptical excision of the mass
Katori H *et al.* [6]	11 years-old	Male	Nasal obstruction Epistaxis	The lateral wall of the nasal cavity	Lobular capillary hemangioma	Elliptical excision of the mass
Virbalas JM *et al.* [7]	12 years-old	Female	Nasal obstruction Epistaxis	Left lateral nasal wall anterior to the middle turbinate	Lobular capillary hemangioma	Endoscopic excision
Ifeacho SN *et al.* [8]	14 years-old	Male	Recurrent epistaxis	Anterior to the right-middle turbinate	Lobular capillary hemangioma	Endoscopic excision
Garefi M *et al.* [9]	9 years-old	Male	Nasal obstruction Epistaxis	The left side of the anterior septum	Lobular capillary hemangioma	Endoscopic excision
Alghamdi B *et al.* [10]	13 years-old	Male	Nasal obstruction Epistaxis	Anterior nasal septum mass	Lobular capillary hemangioma	Endoscopic excision
Mariño-Sánchez F *et al.* [2]	13 years-old	Male	Nasal obstruction Nasal discharge Epistaxis	Inferior turbinate	Lobular capillary hemangioma	Endoscopic excision
	12 years-old	Female	Epistaxis	The anterior septum of the right nasal cavity	Lobular capillary hemangioma	Endoscopic excision
Mills SE *et al.* [1]	10 years-old	Female	Epistaxis	Septum	Lobular capillary hemangioma	Endoscopic excision
Ogunleye AO *et al.* [11]	45 days	Male	Noisy breathing Left nasal cavity swelling	Roof of the left nasal cavity	Lobular capillary hemangioma	Endoscopic excision
Özcan C *et al.* [12]	6 years-old	Female	Nasal obstruction Epistaxis	Right nasal floor	Lobular capillary hemangioma	Endoscopic excision
Benoit MM *et al.* [13]	5 years old	Male	Nasal obstruction Epistaxis	Right nasal septum	Lobular capillary hemangioma	Endoscopic excision
Berlucchi M *et al.* [14]	5 months-old	Male	Nasal obstruction Epistaxis	Left nasal septum	Lobular capillary hemangioma	Endoscopic excision
Kumar MV *et al.* [15]	14 years-old	Male	Nasal obstruction Epistaxis	Left nasal septum	Lobular capillary hemangioma	Endoscopic excision

## Conclusion

Pediatric patients are unlikely to experience nasal LCH. Considering its rare presentation, it is possible to be misdiagnosed. Endonasal endoscopic excision is an effective option to aid both the diagnosis and management. Therefore, LCH should always be considered in the differential diagnosis of vascular lesions within the nose.
